# Stimulation of Chondrogenesis in a Developmental Model of Endochondral Bone Formation by Pulsed Electromagnetic Fields

**DOI:** 10.3390/ijms24043275

**Published:** 2023-02-07

**Authors:** Jake Littman, Roy K. Aaron

**Affiliations:** Department of Orthopedic Surgery, Warren Alpert Medical School of Brown University, Providence, RI 02903, USA

**Keywords:** pulsed electromagnetic field, chondrogenesis, endochondral ossification

## Abstract

Notable characteristics of the skeleton are its responsiveness to physical stimuli and its ability to remodel secondary to changing biophysical environments and thereby fulfill its physiological roles of stability and movement. Bone and cartilage cells have many mechanisms to sense physical cues and activate a variety of genes to synthesize structural molecules to remodel their extracellular matrix and soluble molecules for paracrine signaling. This review describes the response of a developmental model of endochondral bone formation which is translationally relevant to embryogenesis, growth, and repair to an externally applied pulsed electromagnetic field (PEMF). The use of a PEMF allows for the exploration of morphogenesis in the absence of distracting stimuli such as mechanical load and fluid flow. The response of the system is described in terms of the cell differentiation and extracellular matrix synthesis in chondrogenesis. Emphasis is placed upon dosimetry of the applied physical stimulus and some of the mechanisms of tissue response through a developmental process of maturation. PEMFs are used clinically for bone repair and have other potential clinical applications. These features of tissue response and signal dosimetry can be extrapolated to the design of clinically optimal stimulation.

## 1. Introduction

**Figure 1:**
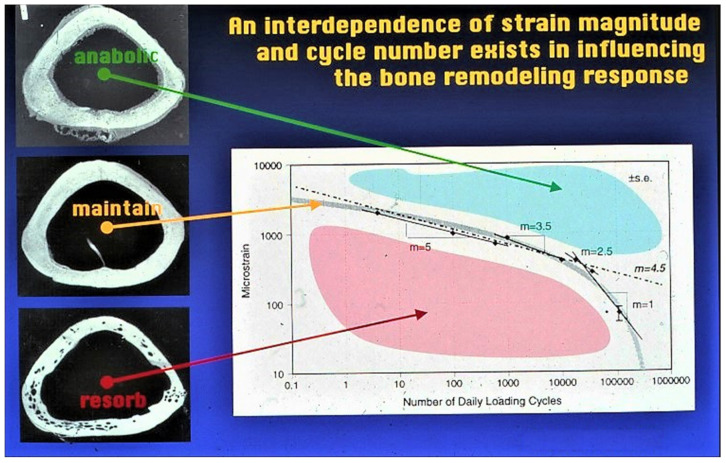
A biomechanical example of how bone responds to physical forces, demonstrating relationships between strain amplitude and frequency on bone mass. An optimal relationship has been found in this model with hypertrophy (blue) and atrophy (red) occurring with increased and decreased strain, respectively. Adapted with permission from Ref. [2]. 1998, *Journal of Orthopaedic Research*.

Musculoskeletal tissues are characterized physiologically by their extracellular matrices (ECM), which are structured to function in a variety of specific physical environments and to perform unique biomechanical functions. In turn, musculoskeletal tissues are responsive to the physical conditions in which they function and the tasks they have to perform. Notable characteristics of the skeleton are its responsiveness to physical stimuli and its ability to remodel in response to changing biophysical conditions, and thereby fulfill its physiological roles of stability and movement [1]. This is observed as homeostasis, hypertrophy, and atrophy (Figure 1). Remodeling the skeleton under a high mechanical load provides the ability to develop strength through hypertrophy to withstand strain.

**Figure 2:**
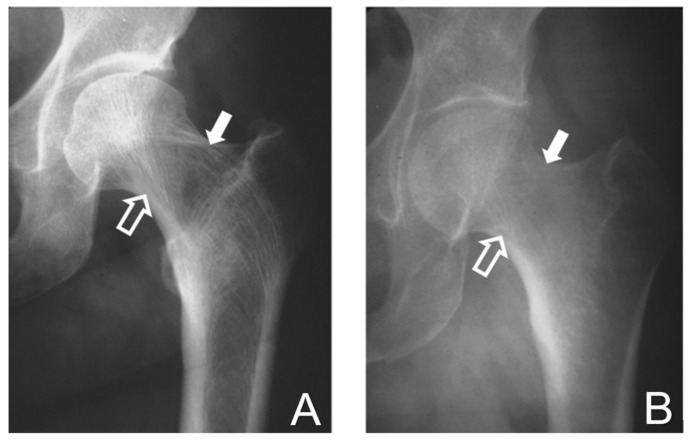
Anteroposterior radiographs of the human proximal femur showing the response to physiological loading (**A**) and osteoporosis (**B**). (**A**) In the normal femoral head, trabeculae align along lines of weight-bearing stress and tissue strain patterns. Compressive trabeculae (open arrows) transmit strain from the joint reactive forces to the medial femoral cortex. Tensile trabeculae (solid arrows) align to resist bending moments due to the offset of the center of rotation of the femoral head from the femoral shaft and transmit bending stress to the lateral femoral cortex. (**B**) In the osteoporotic femoral head, osteoblasts are not responsive to mechanical strain patterns and the trabecular orientation is diminished or lost. Adapted with permission from Ref. [3]. 2022, *Rhode Island Medical Journal*.

The synthesis, organization, and remodeling of ECM are key functions of skeletal cells. One of the most visible manifestations of bone ECM organization is the alignment of trabeculae along lines of physical stress (Figure 2A). Disorders of bone cells can be reflected in insufficient ECM organization, one example of which is osteoporosis, in which the cells do not seem to be responsive to physical stress and do not remodel ECM according to strain patterns (Figure 2B). This often leads to the failure of the bone ECM under physiologic loads to perform its biomechanical functions, and to fractures that often leave in their wake morbidity, disability, and death, and consume substantial amounts of health care resources and costs.

**Figure 3:**
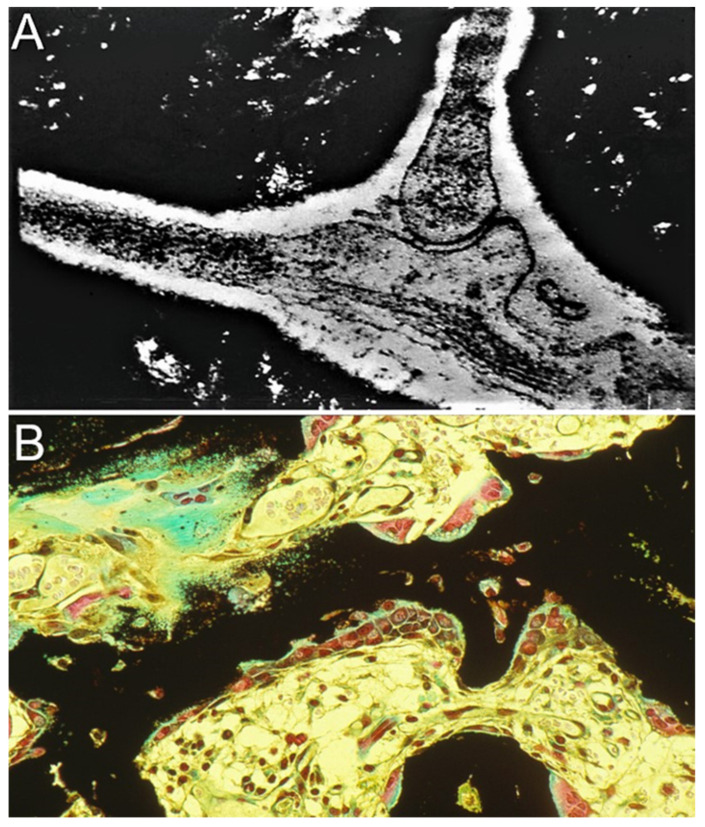
Bone cells functioning in concert in developing and mature bone. Trichrome hematoxylin and eosin stain with silver nitrate showing calcified tissue (black) and unmineralized osteoid (green). (**A**) Bone cells form a functional syncytium in which gap junction channels provide direct intercellular communication pathways for electrical currents, small molecules, and ions, and facilitate the functioning of osteocytes in concert in mature bone to respond to metabolic requirements and mechanical strain. (**B**) Coupled osteoblast and osteoclast remodeling of trabecular bone in an experimental model of endochondral bone formation, resulting in mature trabeculae (40×). Adapted with permission from Ref. [5]. 1988, *Calcified Tissue International*.

Skeletal cells at all stages in their development, including stem cells, osteoblasts, osteoclasts, and osteocytes, exist in micro- and macroenvironments extraordinarily rich in biophysical signaling and intercellular communication, facilitating precursor cell differentiation, coordinated osteoblastic and osteoclastic bone remodeling, and osteocyte responses as a functional syncytium to physiologic demands for calcium (Figure 3). While the most recognizable biophysical force functioning in a signaling capacity is mechanical load, applied mechanical stresses are accompanied by strain-related secondary physical and chemical events that may also function as intracellular communication mechanisms, prominently including fluid flow, hydrostatic pressure, shear stress, and ion-movement-related electrokinetic phenomena, notably streaming potentials [1]. These strain-associated physical events are tightly interrelated, and therefore difficult to isolate. The application of external electromagnetic fields induces an electrical potential in bone that allows for the examination of the responses of bone cells to electrical fields without concordant mechanical strain [4].

This review presents information on cell membrane signal transduction, intracellular message transmission, gene activation, and ECM synthesis in response to externally applied pulsed electromagnetic fields (PEMFs) as isolated physical stimuli. It concentrates on chondrogenesis in endochondral bone formation, a developmental process that facilitates examination of cell differentiation and ECM synthesis in situ. Endochondral bone formation in postnatal life can be understood as a recapitulation, at least in part, of developmental pathways. This involves the re-expression or reinduction of genes that regulate skeletal development and are re-expressed in the growth plate and in fracture repair. We focus on the responses of chondrogenesis to PEMFs, since these physical signals have been shown to promote endochondral bone repair in an orchestrated spatiotemporal manner through a variety of signaling pathways [6]. Understanding the effects of a physical stimulus upon a developmental process in vivo offers insights into cell perception of their physical environment and their responses in terms of gene expression and ECM synthesis. This demonstrates the effects of a physical stimulus on morphogenesis.

## 2. A Developmental Model of Endochondral Bone Formation That Allows for Exploration of Cell Differentiation and Phenotypic Expression of ECM Synthesis in Response to PEMFs

### 2.1. The Demineralized Bone Matrix-Induced Endochondral Bone Formation Model

**Figure 4:**
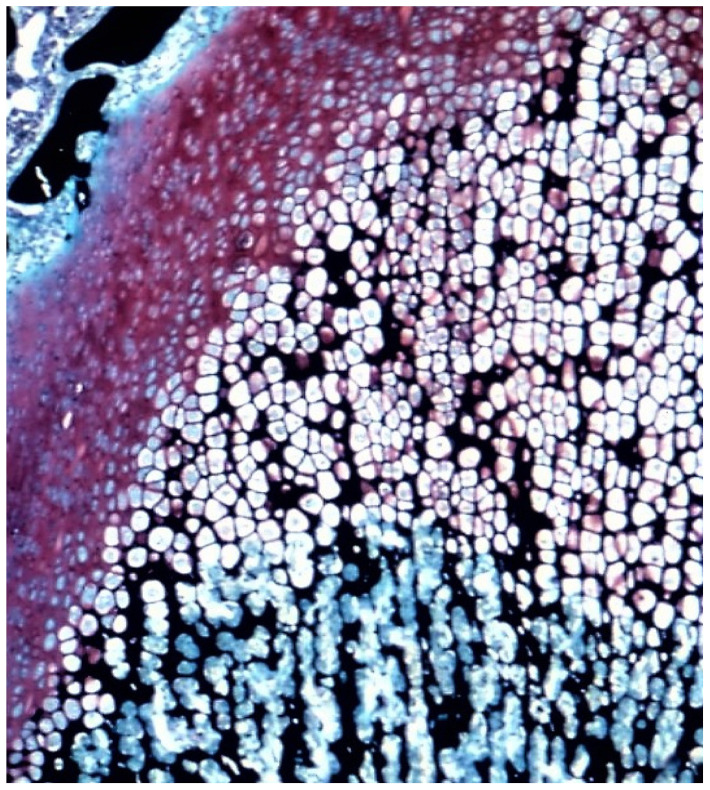
Rat growth plate stained with Safranin O, hematoxylin and eosin, and silver nitrate. The growth plate provides an example of physiological endochondral bone formation. The cartilage precursor (pink Safranin O stain) undergoes calcification (black) and then ingrowth of microvasculature, which produces chondroclasis and replacement by osteoblasts and woven bone (black and blue) (40×). Adapted with permission from Ref. [5]. 1988, *Calcified Tissue International*.

**Figure 5:**
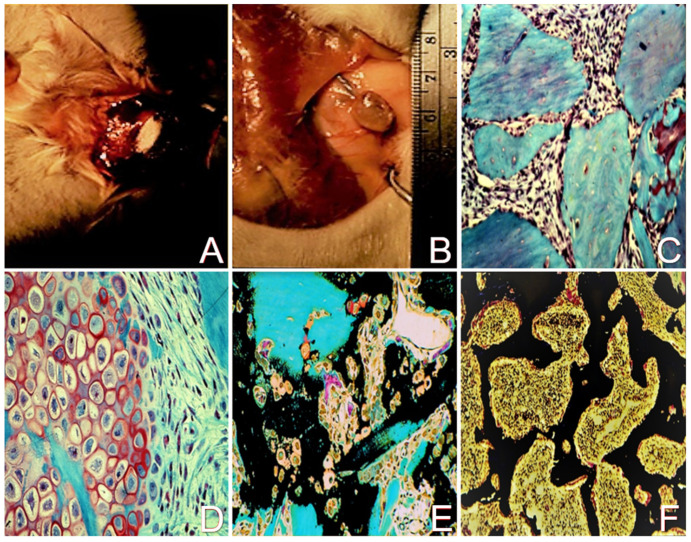
DBM model of endochondral ossification. (**A**) Implantation of DBM granules subcutaneously along the thoracic musculature of CD rats. The implantation of DBM granules results in a temporally predictable sequence of mesenchymal stem/stromal cell infiltration, chondrogenesis, endochondral calcification, and ossification to a mature ossicle. (**B**) An ossicle after 21 days of development. The ossicle contains mature trabeculae and cortex. (**C**) Chemotaxis of infiltrating mesenchymal stem/stromal cells on days 2–4 after DBM (blue) implantation. Homogeneous-appearing gray staining areas are demineralized bone granules (20×). (**D**) Chondrogenesis on days 6–10 after DBM implantation with the demonstration of mesenchymal stem/stromal cell differentiation and several maturing chondrocytes with Safranin O staining ECM (pink) (100×). (**E**) Cartilage calcification and early bone formation (black) on days 10–12 after DBM (blue) implantation (40×). (**F**) Mature trabecular bone (black) on days 14–21 within a surrounding cortex and hematopoietic marrow (20×). All histochemical stains are trichrome, hematoxylin and eosin, Safranin O, and silver nitrate [5]. Adapted from Ref. [8]. 2022. Roy K. Aaron.

Endochondral bone formation is a key process in skeletal morphogenesis. It is the developmental mechanism by which cartilaginous anlage undergoes ossification during embryogenesis, and it is also the mechanism by which the juvenile appendicular skeleton exerts linear growth through the physes (Figure 4). In the adult skeleton, endochondral bone formation is the repair mechanism for fracture healing. In these physiological processes, endochondral bone formation is influenced not only by the physical environment, but also by the hormonal, nutritional, and cytokine environments, and as a consequence, exhibits temporal—and in the case of fracture calluses, spatial—variability. The demineralized bone matrix endochondral ossification (DBM-EO) model, described by Reddi, is highly suitable to study cell responses to PEMFs in the developmental process of endochondral bone formation to physical stimuli because of its predictable and well-described temporal and spatial characteristics of gene expression, cell differentiation, ECM synthesis, chondrogenesis, and bone formation [7]. The predictable cell biology of this model lends itself to exploration of the amplification and acceleration of the cell biology of endochondral bone formation by external stimuli. The DBM-EO model has been described many times, most recently in this journal [1]. In this model, the subcutaneous implantation of DBM granules along the ventral thoracic musculature results in a temporally consistent sequence of mesenchymal stem/stromal cell chemotaxis on days 2–4 after DBM implantation, chondrogenic differentiation by day 6–10, endochondral calcification on days 10–12, and ossification and progressive maturation to an ossicle with cortex, trabecular bone, and marrow elements by days 14–21 after DBM implantation (Figure 5).

### 2.2. Enhancement of Cell Differentiation in the DBM-EO Model by PEMFs

**Figure 6:**
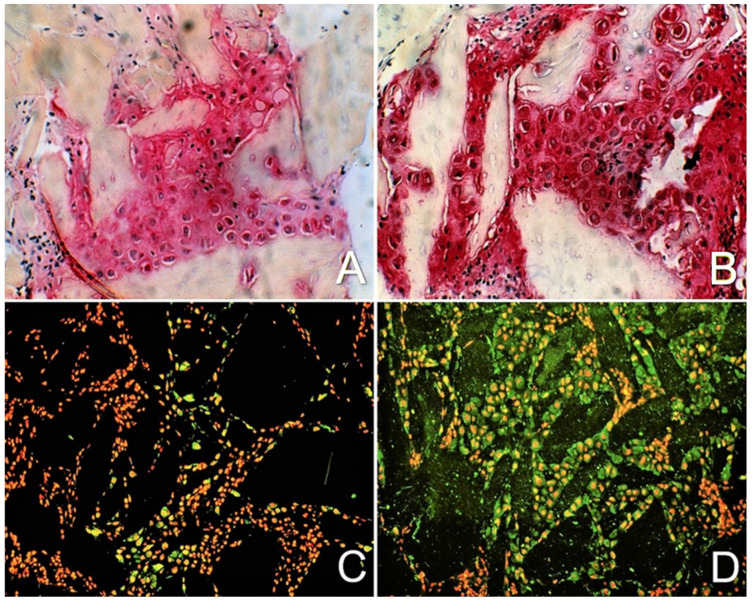
ECM molecules with exposure to PEMFs. (**A**) Trichrome Safranin O histochemistry of unexposed control ossicles. Gray homogeneous areas are DBM particles (40×). (**B**) Histochemistry showing increased ECM deposition of aggrecan (dark Safranin O red staining) in ossicles exposed to PEMFs (40×). (**C**) Immunohistochemistry of unexposed ossicles. Propidium iodide stains the nuclei red. Black areas are DBM particles (20×). (**D**) Immunohistochemistry showing increased ECM deposition of collagen II (green) in PEMF-exposed ossicles (20×). Adapted with permission from Ref. [9]. 2002, *Journal of Orthopaedic Research*.

In detailed studies with the DBM-EO model, evidence for enhanced bone and cartilage differentiation and ECM synthesis by exposure to PEMFs has been presented in morphologic, biochemical, and molecular terms [9]. Exposure of developing endochondral ossification to low-frequency PEMFs enhances chondrocyte differentiation and chondroid ECM synthesis, as evidenced by an increase in mRNA for aggrecan and type II collagen, increases in both radiolabeled sulfate incorporation and glycosaminoglycan content, and the spatial area comprised of cartilage during maximal chondrogenesis (Table 1). The DNA content is not different between the PEMF-exposed and unexposed ossicles, indicating that total cell number is unchanged by PEMF exposure and that the increased number of chondrocytes comprise a greater fraction of the total cell content in PEMF-exposed compared to unexposed control ossicles, supporting the observation of increased cell differentiation by PEMF exposure. Accelerated chondrogenic differentiation by PEMF exposure is demonstrated by the earlier appearance and deposition in the ECM of cartilage-specific proteoglycan (aggrecan). Immunohistochemistry demonstrates an increased accumulation of aggrecan-specific glycosaminoglycans in PEMF-exposed compared to unexposed ossicles. Immunolocalization of proteoglycan and type II collagen demonstrates more chondroid matrix and more mature matrix in PEMF-exposed ossicles (Figure 6). The increased deposition of aggrecan in the ECM of PEMF-exposed ossicles is accompanied by an earlier and quantitatively greater expression of aggrecan and type II collagen genes. A threefold increase in aggrecan gene expression and a twofold increase in type II collagen gene expression are observed in PEMF-exposed compared to unexposed control ossicles (Table 2).

**Figure 7:**
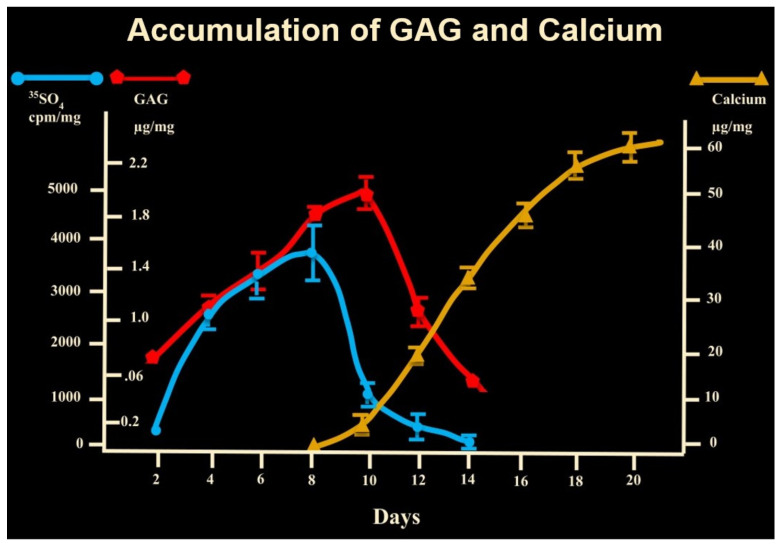
Coordinated proteoglycan and glycosaminoglycan reduction with the onset of calcification. As is typical of physiologic examples of endochondral bone formation, chondrogenesis ceases and chondroid matrix is removed with the onset of calcification. This biphasic process leads to ossification [7].

**Figure 8:**
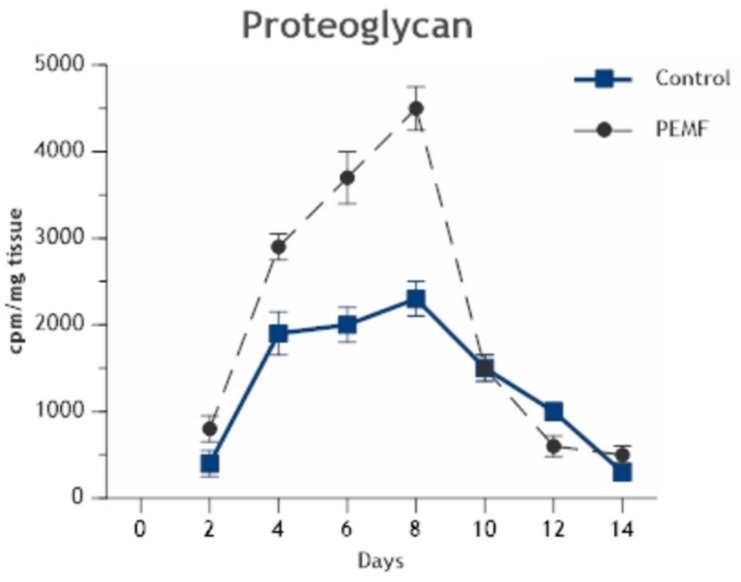
Stimulation of chondrogenesis in endochondral bone formation by PEMFs. Peak of chondrogenesis occurs on day 8 of development in both exposed and unexposed ossicles, with significant increase in aggrecan content in PEMF-exposed ossicles. However, in both exposed and unexposed ossicles, proteoglycan content rapidly decreases with the onset of calcification. With PEMF stimulation, there is no disorganization of temporal sequences of chondrogenesis. Adapted with permission from Ref. [11]. 2006, *Annals of the New York Academy of Sciences*.

Other details of the acceleration of endochondral bone formation by PEMFs are important to note. With the transition of chondrogenesis to bone formation, the content of proteoglycan and glycosaminoglycan in the ECM decreases markedly as calcification is initiated (Figure 7). This is characteristic of all forms of endochondral ossification as cartilage is calcified and replaced by bone. The DBM-EO model exhibits this transition in a predictable temporal sequence, at days 8–10 of ossicle development, as calcification proceeds. Importantly, PEMF stimulation of chondrogenesis accentuates proteoglycan synthesis but does not disorganize the temporal or quantitative processes of proteoglycan removal with the onset of calcification and replacement with bone (Figure 8) [7,9]. A second important observation is the cellular loci of stimulation of endochondral bone formation by PEMFs. In addition to the stimulation of chondrogenesis by chondrocytes, PEMFs stimulate the differentiation of mesenchymal stem/stromal cells to chondrocytes. The locus of this stimulation of chondrogenic differentiation resides in the mesenchymal phase of DBM-EO. Measurements of chondrogenesis on day 8 of ossicle development have demonstrated that exposure to PEMFs during either the mesenchymal or chondrogenic phases stimulate chondrogenesis to similar degrees. Further, developing ossicles exposed during the mesenchymal phase only exhibit trabecular volume and maturation equivalent to that seen in ossicles stimulated throughout their developmental sequence or during bone formation. These observations indicate that exposure to PEMFs during the mesenchymal phase augments the downstream formation and maturation of trabecular bone. Thus, it appears that exposure to PEMFs during the mesenchymal stage produces an increase in both bone and cartilage formation, whereas exposure during the cartilage phase increases chondrogenesis but not the formation of trabecular bone. These observations collectively suggest that the mesenchymal stem/stromal cell pool is most responsive to PEMF exposure for enhanced bone formation [9].

The sensitivity of specific cells to PEMFs has been confirmed by a substantial number of studies of PEMFs upon both mesenchymal stem/stromal cells (MSCs) and chondrocytes. One study reviewed the responses of MSCs to a variety of biophysical stimuli that could be characterized as cellular signals including hydrostatic pressure and fluid flow shear stress, substrate topography and induced strain, and electromagnetic fields, and concluded that MSCs were responsive to all these physical stimuli most likely through stretch-activated or voltage-activated membrane cation channels that open due to membrane deformation or exposure to electromagnetic fields and fluid streaming potentials. In this construct, several biophysical signaling events may act synergistically to regulate MSC differentiation [12]. In a focused study of the effect of PEMFs on human bone-marrow-derived MSCs, increased osteoblastic gene expression and markers of osteogenic ECM in response to a specific PEMFs were observed [13]. PEMFs have been shown to enhance both the osteogenic and chondrogenic differentiation of MSCs, most likely through adenosine stimulation [14].

### 2.3. TGF-β Signaling under PEMF Stimulation in the DBM-EO Model

Members of the transforming growth factor beta (TGF-β)/bone morphogenic protein (BMP) family, notably TGF-β 1–3 and BMP 2 and 4, upregulate cartilage and bone morphogenesis. They are expressed during chondrogenesis in skeletal development and are re-expressed in the growth plate and in fracture repair. Several laboratories have demonstrated that exposure to electromagnetic fields upregulates members of the TGF-β/BMP family in isolated cell cultures of MC3T3, MG63, and human fracture nonunion cells [15,16,17]. While in vitro cell systems offer the opportunity to study isolated cellular responses, in vivo studies with a highly inbred strain of animals avoid artifacts caused by tissue culture, maintain cells and intercellular relationships within the ECM, and provide the ability to observe developmental processes, such as endochondral ossification, that are translationally relevant to human pathophysiology in embryogenesis, growth plates, and fracture repair. Developmental systems are also vastly more complex than isolated cell cultures, involving exquisite timing of cell receptivity to applied stimuli. We have just reviewed the enhancement of chondrogenesis and endochondral ossification in the DBM-EO model by PEMFs and the dependency of those enhancements upon chondrocyte differentiation of the progenitor mesenchymal stem/stromal cell pool. In subsequent studies with the same developmental model of endochondral ossification, it was demonstrated that exposure to PEMFs also results in an increase in TGF-β mRNA and protein synthesis above constitutive levels associated with chondrogenic differentiation [10]. The ability to upregulate TGF-β with a physical agent coincident with enhanced mesenchymal and chondrocytic differentiation and enhanced cartilage and bone formation has significant implications for stimulating skeletal repair and for tissue engineering of bone.

**Figure 9:**
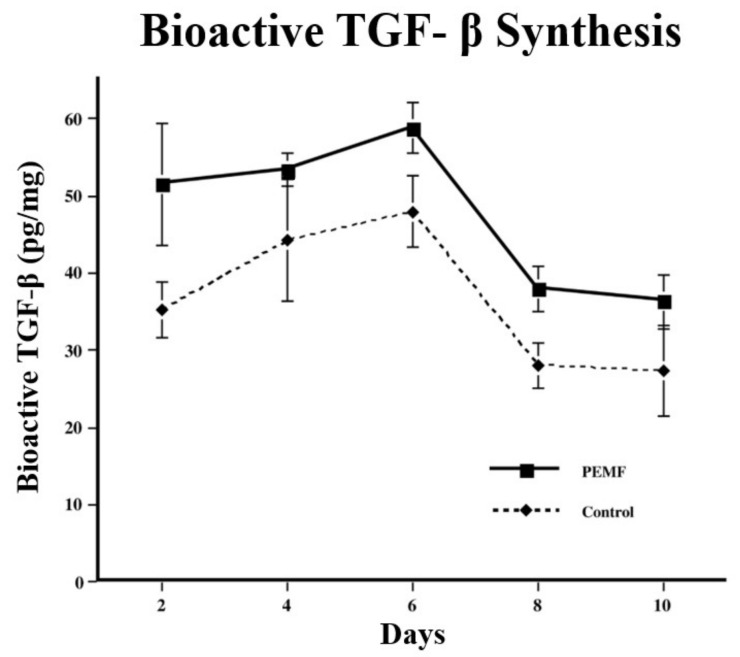
Increase in bioactive TGF-β protein (pg/mg ossicle) with PEMF stimulation. Significant increases are seen at days 2, 6, and 8 of development (<0.05). TGF-β protein is increased in pre-chondrogenic and early chondrogenic tissue with a decrease on day 8 just prior to matrix calcification. The constitutive pattern of TGF-β expression is increased but not disorganized. Adapted with permission from Ref. [11]. 2006, *Annals of the New York Academy of Sciences*.

**Figure 10:**
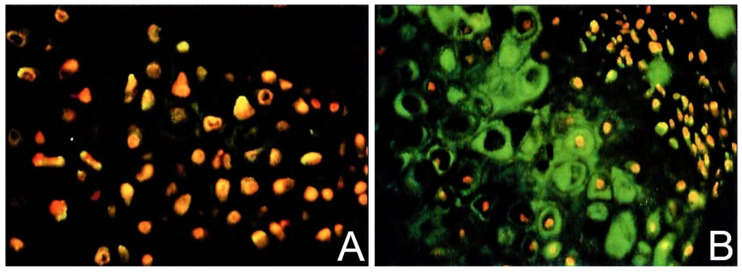
Immunohistochemistry of TGF-β1 synthesis with PEMF exposure. (**A**) Unexposed ossicles showing propidium iodide staining nuclei (red) and no TGF-β1 staining in the ECM (40×). (**B**) PEMF-stimulated ossicles showing TGF-β1 synthesized by chondrocytes in areas of cartilage formation (green) rather than by mesenchymal stem/stromal cells (40×). Adapted with permission from Ref. [10]. 2002, *Journal of Orthopaedic Research*.

**Figure 11:**
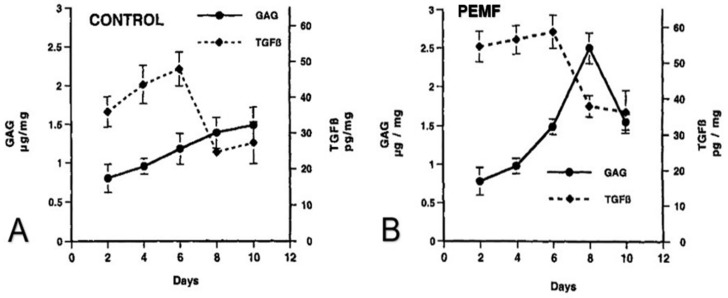
Coordinated chondrogenesis and TGF-β. (**A**) In control (unexposed) ossicles, TGF-β protein synthesis is coincident with glycosaminoglycans (GAGs) preceding and during chondrogenesis and decreases with the onset of calcification. (**B**) In PEMF-exposed ossicles, an increase in glycosaminoglycans (GAGs) is seen with chondrogenesis. TGF-β protein is markedly increased and then decreases with the onset of matrix calcification. Adapted with permission from Ref. [10]. 2002, *Journal of Orthopaedic Research*.

Using the model of DBM-EO and a PEMF consisting of a pulse-burst magnetic field of 4.5 ms duration repeated at 15 Hz with a peak magnetic field of 16 G, the synthesis of TGF-β in chondrogenesis was described [10]. Chondrogenesis was characterized by the incorporation of radiolabeled macromolecular sulfate for proteoglycan and with a colorimetric assay with dimethylmethylene blue for glycosaminoglycans. Biologically active TGF-β protein concentrations were determined by ELISA. Immunostaining-characterized tissue distribution of TGF-β. TGF-β, proteoglycan, and type I collagen mRNA were measured by Northern blot analysis. Significant increases in TGF-β protein concentration were observed in PEMF-stimulated ossicles compared to unstimulated control ossicles from the mesenchymal stage (day 2) through chondrogenesis and early calcification (days 8–10) (Figure 9). The normal physiologic peak of TGF-β during early chondrogenesis (day 6 of development) was observed also in PEMF-stimulated ossicles and was quantitatively increased. Maximal TGF-β synthesis was observed at day 6 of development during chondrocytic differentiation and early chondrogenesis (Table 3). Immunohistochemistry revealed that the TGF-β was in association with chondrocytes rather than mesenchymal stem/stromal cells (Figure 10). Importantly, with the cessation of chondrogenesis and the beginnings of early calcification, glycosaminoglycans were removed and TGF-β protein concentration declined, indicating that PEMF exposure increased TGF-β without disorganizing its physiology, and also demonstrating the establishment of temporally appropriate concentrations of TGF-β associated with stimulation and cessation of chondrogenesis (Figure 11). The relationship of TGF-β to Smads has been well documented, but that relationship is beyond the scope of this review.

### 2.4. PEMF Influences on Signal Transduction, Intracellular Messaging, and Activation of TGF-β

With the demonstration of the effects of PEMFs upon ECM synthesis both in vivo and in vitro, the promotion of cell differentiation by PEMFs, and the role of TGF-β, recent attention has turned to the stimulation by PEMFs of mechanisms of membrane reception, signal transduction, and messaging of the cellular response, culminating in gene activation.

#### 2.4.1. Signal Transduction

Transmembrane reception of physical signals is incompletely understood. In fact, there may be duplicative processes since nature seems to favor redundancy. Mechanical, fluid flow-related, and electrokinetic physical phenomena have been shown to activate a variety of transmembrane channels serving as signals to cells about the state of their biophysical environment and eliciting responses from cells, either as paracrine signaling or synthesis and resorption of ECM structural molecules. Voltage-gated transmembrane channels may be activated by PEMFs and are mediated by transmembrane calcium flux. Another transmembrane signaling complex shown to be activated by PEMFs is transient receptor potential (TRP) cation channels, which appear to be signal-specific [18]. Exposure to PEMFs has also been shown to activate a calcium–mitochondrial axis [19]. A series of studies using pharmacological models has demonstrated that PEMFs activate adenosine receptors, particularly A2A, which in turn is stimulatory to chondrogenesis [20]. PEMF exposure induces a significant increase in adenosine A2A and A3 receptor density on the cell membranes of chondrocytes, osteoblasts, and synoviocytes, and synergizing with TGF-β, participates in chondrogenic differentiation. The result of the adenosine signaling by PEMFs is to instruct cells to remodel bone or cartilage ECM, enhancing the ability of skeletal tissues to respond to changing physical environments and biomechanical needs, and facilitating repair [4,14].

#### 2.4.2. Intracellular Messaging

Several signaling pathways have been implicated in the osteoblast and chondrocyte response to PEMFs and in bone formation in skeletal repair [6]. These pathways can only be mentioned for completeness here. PEMFs have been shown to elevate the expression of voltage-gated transmembrane calcium channels with signal transduction through elevated intracellular calcium. Another important pathway shown to be activated by exposure to PEMFs in osteoblasts is the canonical Wnt/β-catenin pathway and the differentiation-related genes in osteoblasts for alkaline phosphatase, osteocalcin, collagen 1, and Runx2. A third pathway, and one that is important in osteogenic differentiation, is the mitogen-activated protein kinases (MAPKs), which have been shown to be activated by PEMFs. The fourth pathway of significance, especially in developing skeletal systems, is the TGF-β/BMP gene family. Several other intracellular second messengers have been implicated in transmitting PEMF signals, including cAMP and Notch [4,21]. Of particular interest in cell differentiation and chondrogenesis under PEMF stimulation are the complex interactions among the MAPKs and TGF-β. PEMFs have been shown to induce the activation of protein kinase B and MAPK signaling cascades in mesenchymal stem/stromal cells [22]. Adenosine receptors activate MAPKs and may be a pathway of intracellular messaging from PEMFs.

**Figure 12:**
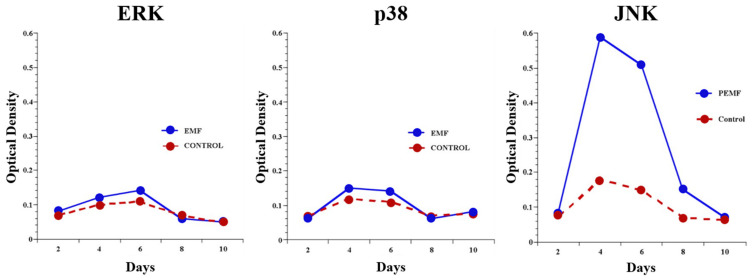
MAPK phosphorylation with PEMF exposure. Western blot analysis of the MAP Kinase proteins JNK, ERK, and p38, as well as their phosphorylated counterparts, indicates that of the three, only JNK is affected by exposure to PEMFs. Basal levels of JNK protein are expressed consistently with normal development; however, the phosphorylated protein is significantly elevated by a factor of greater than 3-fold. Adapted from Ref. [23]. 2004. Roy K. Aaron.

MAPKs are serine/threonine kinases that play important roles in a wide range of pathophysiological processes, including gene expression, proliferation, differentiation, and apoptosis. The MAPK pathways are responsive to external signals and allow cells to interpret those signals and to respond rapidly. The MAPK family is comprised of three main subfamilies: extracellular-regulated kinases (ERKs), Jun N-terminal kinases (JNKs), and p38 mitogen-activated protein kinases. ERKs are activated by mitogenic stimuli; JNK and p38 MAPK are activated primarily by environmental physical stimuli such as PEMFs and are referred to as stress-activated protein kinases. They facilitate the activation of nuclear transcription factors in response to extracellular signals and they signal chondrocytes to react to environmental stresses and are involved in the response to PEMFs. MAPK pathways control the cell response to changes in the extracellular environment through the regulation of transcription factors in the nucleus [22]. To transmit extracellular signals to the nucleus, MAPK pathway elements such as ERK and JNK must be activated by phosphorylation and translocate to the nucleus. One study with the DBM-EO model has shown increased phosphorylation of JNK in response to PEMF stimulation [23]. The electromobility shift assay (EMSA) and Western blots were used to examine the unphosphorylated and phosphorylated constituent elements of MAPK. Of the three MAPK constituents, only JNK phosphorylation was increased by exposure to PEMFs (Figure 12). Another study has shown that PEMF exposure activates protein kinase B and the MAPK signaling cascade in mesenchymal stem/stromal cells and correspondingly upregulates the ECM molecules osteocalcin, alkaline phosphatase, and collagen I [24]. Extensive crosstalk exists between MAPK and TGF-β signaling, of relevance to tissue responses to PEMFs. Studies of the MAPK family in several biological systems have shown that the MAPK and TGF-β signaling pathways interact with each other and have a synergistic effect on the secretion of other growth factors and cytokines. TGF-β has been shown to activate all ERK, p38 MAPK, and JNK MAPKs in numerous cell types, but perhaps the best-characterized interaction between TGF-β and MAPK signaling involves the JNK and p38 MAPK signaling cascades [22]. A reciprocal activation of MAPK, particularly JNK and p38, by TGF-β comprises some of the complex interactions between the MAPK and TGF-β that are dependent upon cell type and receptivity, and physiological stage of development.

#### 2.4.3. Activation of TGF-β

**Figure 13:**
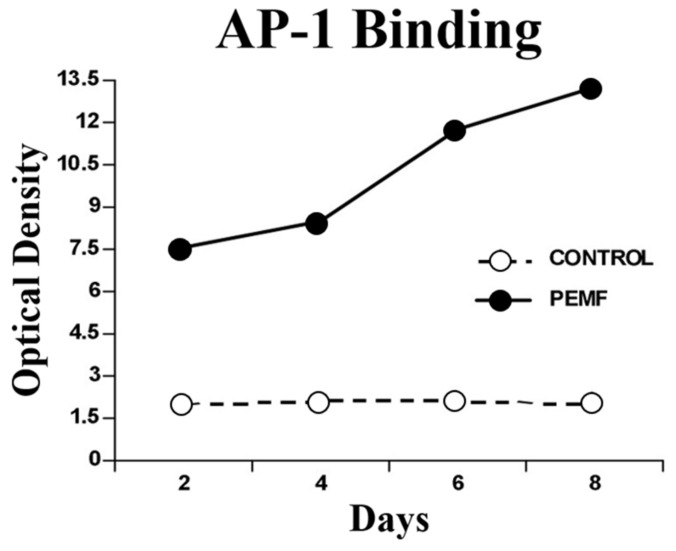
PEMFs increase AP-1 binding to TGF-β. Electromobility shift assay (EMSA) of nuclear extracts demonstrating binding of the transcription factor AP-1. At all time points, the binding in the PEMF-exposed ossicles was significantly higher, indicating an increased level of transcriptional activity. Adapted from Ref. [23]. 2004. Roy K. Aaron.

**Figure 14:**
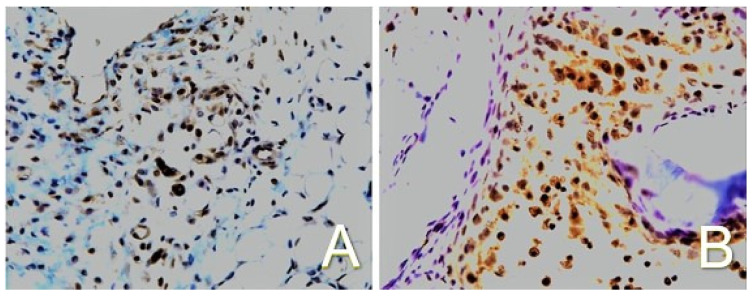
Immunohistochemistry with goat polyclonal anti-c-Jun antibody conjugated to horseradish peroxidase. Diaminobenzidine was used as a chromogen and produces a brown color, demonstrating expression of c-Jun upon a background of collagenous connective tissue (blue and purple). (**A**) Without PEMF exposure showing minimal c-Jun activity (20×). (**B**) With PEMF exposure demonstrating active c-Jun expression (20×). Adapted from Ref. [8]. 2022. Roy K. Aaron.

Several transcription factors serve as substrates for, and are activated by, MAPKs. One of importance in chondrogenic differentiation is activating protein-1 (AP-1), which regulates early response gene expression to a variety of external stimuli and controls a number of cellular processes, including differentiation. AP-1 serves as a transcription factor for TGF-β and enhances TGF-β expression in response to PEMF exposure. AP-1 is a complex of the DNA binding proteins of the Jun and Fos families of early response genes c-Jun and c-Fos. JNK is the only MAPK that phosphorylates c-Jun, a main component of AP-1 complexes [22]. Exposure to PEMFs increases the binding of AP-1 and the expression of TGF-β coincident with chondrogenesis. Electromobility shift assay of nuclear extracts has demonstrated binding of the transcription factor AP-1 at all developmental time points in both PEMF-exposed and unexposed ossicles; however, at all time points, the binding in the PEMF-treated ossicles was significantly higher, indicating an increased level of transcriptional activity (Figure 13). In contrast, the unexposed population exhibits the expected basal activity levels. Increases in tissue c-Jun have been observed in the same developmental model of endochondral bone formation by exposure to PEMFs, supporting the observation of increased AP-1 activity in response to PEMFs (Figure 14). Activated c-Jun can then promote the binding of AP-1 to specific recognition sites, including on TGF-β. Increases in the transcription of TGF-β mRNA coincident with chondrogenesis suggest that the stimulation of TGF-β expression may be a mechanism through which PEMFs affect complex tissue functions such as cell differentiation and through which the effects of PEMFs may be amplified.

## 3. PEMF Dosimetry in the Regulation of Cell Responses

Dosimetry of applied PEMFs has to be considered from the perspectives of tissue specificity and signal specificity. Tissue specificity refers to the ability of cells to respond to the physical stimulus. In terms of a developmental biological process, it has been well shown that the receptivity of cells to regulatory factors is often sequential and that the timing of cell receptivity and exposure to stimuli must be coincident for an appropriate change in tissue morphogenesis to occur. Different responses (proliferation, differentiation, etc.) can be expected from different cells, or the position in the cell cycle, when exposed to physical stimuli to which they are responsive. Signal specificity refers to the appropriate dosing and timing of application of physical stimuli. A crucial issue in eliciting cell reactions from physical stimuli, including PEMFs, is the complex web of signal dosimetry. Dosimetry can be expressed as signal amplitude, frequency, duration of exposure, on–off cycle, and a variety of signal shapes and configurations. The literature is vast, reporting changes in tissue morphogenesis with a wide variety of PEMF signals, often with little empirical evidence.

Two reviews have summarized the effects of a variety of PEMFs on experimental and clinical models [11,25]. It has been claimed that only a few low-frequency PEMF signals are supported by clinical evidence. Active PEMF signals are described typically as trapezoidal or sawtooth waves, with magnetic field peak intensities spanning from 1.2–2 mT and signal repetition frequencies between 15–75 Hz [14]. Others report a wider range of active signals from 0.1–3 mT and 7.5–75 Hz [24]. Presented here are a sampling of studies demonstrating enhanced morphogenesis with a variety of signal dosages.

A mathematical model characterized the responses on single-cell and cell-population scales to an applied electrical field and compared the model to experimental results with human MSCs stimulated with a novel electrical field device [26]. The model confirms the effects of the electrical field on the cell population and defines the time scale of differentiation as occurring gradually. Other studies with MSCs have used a daily stimulation for 28 days with a 300 μs quasi-rectangular pulse at a rate of 7.5 Hz, and have demonstrated early proliferation and subsequently the appearance of early osteogenic genes, including Runx2/Cbfa1 and alkaline phosphatase, as well as alkaline phosphatase protein and alizarin red staining, indicating augmented osteogenesis [13]. In addition to studies on MSC differentiation, the effects of PEMFs on chondrocytes have been described, usually using in vitro models. Using human arthritic chondrocytes obtained from total knee arthroplasty procedures, cell viability was found to be highly dose-dependent, with a 1.95 μT, 0.1 Hz field applied for 60 min/day for 3 days being most effective and a 1.3 μT, 10 Hz field applied for the same 60 min/day being ineffective, demonstrating the effects of field dosimetry on cell responsiveness [27]. A study with in vitro culture of chondroprogenitor cells compared the effects of PEMFs with those of TGF-β in the stimulation of chondrogenic differentiation [28]. Cells were stimulated with a single exposure of 2 mT, 15 Hz, and pulse duration 6 msec for 10 min at day 0 of differentiation or every three days (7 exposures in 21 days) and the effects on differentiation were found to be similar to the TGF-β positive controls with the upregulation of Sox9, Col2, and aggrecan mRNA after 21 days of culture. In an alternative approach, different from the administration of repetitive exposures to PEMFs, optimal chondrogenic outcome was reported in response to single, 10 min, low-intensity (2 mT) exposure at 15 Hz frequency administered at the onset of chondrogenic induction [18]. The same group reported modulation of the MSC chondrogenic secretome with a 10 min application of a PEMF of 0.5–4 mT of 6 ms repeated at a frequency of 15 Hz [29]. MAPK activation and upregulation of osteocalcin, alkaline phosphatase, and collagen I were reported with a 26 Hz PEMF, although amplitudes and other parameters were not reported [24]. PEMFs have been shown to increase proteoglycan synthesis in articular cartilage explants dependent upon field intensity, frequency, and exposure duration [30]. Field intensity was shown to be a significant factor from 0.5–2.0 mT; frequency was significant between 2–110 Hz, with minimal differences among 4 doses; exposure length produced significant increases in proteoglycan from 4–24 h. Longer exposures were not tested. In this study, each parameter was examined independently rather than as a flexible matrix.

## 4. Discussion

Skeletal morphogenesis is highly responsive to its physical environment and responds to a variety of physical cues. Bone and cartilage cells have many mechanisms by which to sense these cues, transmit information, and activate a variety of genes to remodel their ECM and create secondary paracrine signaling. The ability of cells to perceive their physical environment is complex, with several potential transmembrane receptors and an interactive web of intracellular mediators and transcription factors influencing a large number of genes controlling both structural molecules for morphogenesis and soluble molecules for paracrine signaling.

While in vitro studies of cells provide opportunities for studying detailed cell responses to physical stimuli, in vivo studies with developmental models, such as endochondral bone formation, permit the exploration of cell differentiation and signaling in systems that are translationally relevant to the physiological events of embryogenesis, skeletal growth, and fracture repair [1]. The developmental nature of endochondral bone formation permits the examination of the effects of PEMFs on the timing and magnitude of cell differentiation and ECM synthesis in both cartilage and bone. A corollary to these observations is the receptivity of cells at varying stages of maturation to paracrine and physical signaling, including PEMFs. The reactions of biological tissues to environmentally applied physical forces are extremely complex and are dependent upon the specific tissue being exposed and the dosimetry of the applied force. With regard to PEMFs, the dosimetric matrix includes signal amplitude, frequency, duration of exposure, on–off cycle, and signal configuration. Cell and tissue responses depend upon the receptivity of the cell to the PEMFs, so the timing of exposure to PEMFs is critical to produce changes in morphogenesis. A developmental model such as presented here characteristically contains cells that are receptive to stimuli at stages in their development, and if not exposed to relevant stimulus during the period of receptivity, do not proliferate or differentiate, and morphogenesis is disrupted. The study, of course, has limitations as well. The model is artifactual and the temporal sequences and magnitudes of responses observed may be particular to this model and not exactly extrapolatable to other forms of endochondral bone formation. While growth plates have a linear spatial orientation, fracture callus, while exhibiting similar biology, is spatially more diverse. The DBM-induced model presented here also has a particular set and timing of responses to the DBM and to the PEMF and may not be generalizable to other forms of endochondral bone formation regulated by different hormonal or biomechanical patterns.

This review explores the regulation of cell differentiation in the developmental context of endochondral bone formation by an external PEMF, which has wide clinical applications to the stimulation of skeletal repair. In this model of endochondral ossification, TGF-β is constitutively coexpressed with augmented and accelerated proteoglycan and type II collagen synthesis during chondrogenesis and chondrocyte hypertrophy [4]. PEMF exposure produces the upregulation of TGF-β and ECM molecules with preservation of their temporal peaks and cessation during maximal chondrogenesis and subsequent cartilage replacement, respectively. These observations suggest that PEMF exposure increases—but does not disorganize—endochondral ossification, indicating the potential for PEMFs to augment stem cell differentiation, chondrogenesis, and endochondral bone formation, and potential clinical applications for fracture repair and tissue engineering.

As has been expressed by Caplan and Goldberg, “A consolidating paradigm of regeneration is thought to be the recapitulation of the embryonic events of development in the context of the adult environment. Obviously, this recapitulation requires tissue-specific, biologic and physical cues” [31]. The stimulatory effects of PEMFs, a physical cue, upon the developmental features of endochondral bone formation have been presented. Endochondral bone formation presents opportunities for examining the effects of PEMFs upon morphogenesis and some of the mechanisms thought to be activated in the process of stimulation. This information can be used to optimize the interaction of PEMFs and morphogenesis, and to make physical stimulation in general more effective in clinical applications [7].

## Figures and Tables

**Table 1:** Indices of chondrogenesis on day 8 with PEMFs. On day 8 of development, during maximal chondrogenesis, ossicles exposed to PEMFs demonstrated a two-fold increase in sulfate incorporation into proteoglycan and glycosaminoglycan content compared to unexposed ossicles. The spatial area of ossicle comprised of cartilage and the number of chondrocytes were increased by PEMF exposure. The glycosaminoglycan (GAG) contents expressed per unit area or per chondrocyte and the chondrocyte:matrix ratios were not different in control vs. exposed ossicles, indicating the production of more chondrocytes in PEMF-exposed ossicles rather than an increase in proteoglycan synthesis per chondrocyte. Adapted with permission from Ref. [10]. 2002, *Journal of Orthopaedic Research*.

	Control	PEMF	Percent Difference	*p*-Value
mRNA aggrecan	6.1	22.5	269	0.02
mRNA type II collagen	11.8	21.9	86	0.05
^35^SO_4_ incorporation (cpm/mg)	2166 ± 387	4448 ± 293	105	0.005
GAG content (μg/mg)	1.4 ± 0.2	2.5 ± 0.2	79	0.01
Cartilage area (mm^2^)	24 ± 2.1	148 ± 11.7	517	0.001
Chondrocytes (n)	701 ± 227	3582 ± 675	411	0.005
Chondrocyte/cartilage	29.2	24.2		n.s.

**Table 2:** Increased expression of cartilage genes with PEMF exposure. Gene expression normalized to GAPDH shows a 3-fold increase in aggrecan expression on both days 6 and 8 of development and a 2-fold increase in collagen II on day 8 with PEMF exposure. The aggrecan gene typically resolves into 2 bands, which is why both the 7.4 and 8.0 kb variants were seen. Both variants exhibited a 3-fold increase in expression with PEMF exposure. Adapted with permission from Ref. [9]. 2002, *Journal of Orthopaedic Research*.

		Aggrecan (kb)		α_1_ (II) Collagen
		8.0	7.4	
Day 6	Control	3.9	3.9	0
	Exposed	13.3 *	13.3 *	0
Day 8	Control	6.2	6.1	11.8
	Exposed	17.8 *	22.5 *	21.9 *

* *p* < 0.05 compared to respective controls.

**Table 3:** Quantitative increases in TGF-β synthesis with PEMFs. On day 6 of development, during the peak TGF-β production, the increase produced by PEMF stimulation was 0.08 pg/pg DNA. Overall, PEMF exposure produced significant increases in TGF-β protein, mRNA, and immunopositive cells compared to unexposed control ossicles. Adapted with permission from Ref. [10]. 2002, *Journal of Orthopaedic Research*.

	Control	PEMF	Percent Difference	*p*-Value
mRNA (OD/GAPDH)	21.0 ± 4.4	54.1 ± 0.7	158	0.03
Protein (pg/ng)	47.9 ± 4.7	58.8 ± 3.2	23	0.05
Immuno (+) cells (n)	121.8 ± 43.8	540.0 ± 118.7	343	0.001

## Data Availability

Not applicable.
